# Role of gene interactions in the pathophysiology of skeletal dysplasias: A case report in Colombia

**DOI:** 10.1016/j.jgeb.2023.100350

**Published:** 2024-02-01

**Authors:** Nathalie Yepes Madrid, Lina Johanna Moreno Giraldo

**Affiliations:** a Pediatric Specialization Resident, Universidad Libre Cali, Colombia; b Universidad Libre Cali Sectional, Colombia; c Pediatric Research Group (GRINPED), Colombia; d Neurogenetic and Metabolic Diseases Research Line, Colombia

**Keywords:** Interaction, Genes, Genotype, Phenotype, Skeletal dysplasia, Medical advice

## Abstract

**Background:**

Genome association studies have shown that gene-gene interactions or epistasis play a crucial role in identifying the etiology, prognosis, and treatment response of many complex diseases beyond their main effects. Skeletal dysplasias are a heterogeneous group of congenital bone and cartilage disorders with a genetic and gen-gen interaction etiology. The current classification of skeletal dysplasias distinguishes 461 diseases in 42 groups, and the incidence of all skeletal dysplasias is more than 1 in every 5000 newborns. The objective is to present the case of a patient with four variants that generates gen-gen interactions in the skeletal dysplasia.

**Case presentation:**

A 1-year-old male patient was diagnosed with skeletal dysplasia based on prenatal ultrasound showing micromelia and pyelocalyceal dilation. Postnatal physical examination revealed body disproportion and involvement of other organs and systems.

**Materials and Methods:**

A sequencing study and deletions/duplications analysis were performed for 358 candidate genes associated with skeletal dysplasia.

The GeneMANIA interface was used to evaluate the expression network of genes associated with each other for the gen-gen interaction.

**Results:**

Four pathogenic variants were obtained two heterozygous variants with pathogenic significance in *SLC26A*, one heterozygous pathogenic variant in *CLCN7* and another heterozygous pathogenic variant in *CEP120*.

The GeneMANIA interface reveals 77.64% physical interactions, 8.01% co-expression, 5.37% prediction, 3.63% co-localization, 2.87% genetic interactions, 1.88% route of action, and 0.60% shared protein domains.

**Discussion and Conclusions:**

These results suggest that the interaction between these genes affects the activity of the inorganic anion exchanger, leading to disorganization of collagen fibers, early mineralization, and decreased assembly of fibronectin in the bone extracellular matrix. Identifying gene-gene interactions is a fundamental step in understanding proper cell function and thus understanding the pathophysiology of many complex human diseases, improving diagnosis, and the possibilities of new personalized therapies.

## 1 Background

One of the primary objectives of contemporary genetic research is to comprehend the genetic underpinnings of heritable traits and phenotypes, including disease susceptibility. Since the advent of genome-wide interaction studies, genome-wide interaction studies have identified genetic associations for hundreds of phenotypes. Demonstrating that gene-gene (GG) interactions, also known as genetic epistasis, play a crucial role in identifying the etiology, prognosis, and response to treatment of many complex diseases beyond the main effects. Current interaction detection methods only account for single interactions between major SNPs, leaving the extent to which genetic interactions impact observed phenotypes uncertain. In published studies, GG factors include gene expressions, methylation, SNPs, as well as several other types of omics measurements, for which the most widely used in the literature are genome-wide association studies (GWAS), successfully identifying alleles of risk for complex diseases by an association of SNPs with disease-related phenotypes.^1^, ^2^, ^3^, ^4^, ^5^

Gene variations resulting from epistasis can affect more than one system. In the case of the musculoskeletal system, patients are characterized by short stature, impaired ossification, gait, and orthopedic problems, known as musculoskeletal dysplasia (SD, Skeletal dysplasia). There are more than 461 disorders that are classified as SD, which can be classified into 42 distinct groups, of which 5 % are related to congenital defects.^6^, ^7^, ^8^, ^9^, ^10^, ^11^

In the latest epidemiological studies worldwide, it has been reported that the incidence of SD is from 2.3 to 7.6 per 10,000 live births, of these, it is known that osteogenesis imperfecta (OI), the most common pathological entity of SD, is 1 in 15,000 live births worldwide. However, the true worldwide incidence rate may be higher since SD encompasses both viable and life-limiting fatal disorders. Therefore, genomic, exomic, and GG interaction studies are crucial for timely diagnosis, management, and prognosis of these patients.^9^, ^11^, ^12^, ^13^, ^14^, ^15^, ^16^, ^17^

The objective is to present the case of a patient with four variants that generates gen-gen interactions in the skeletal dysplasia.

## 2 Consent

After obtaining consent from the mother, we got approval from the medical ethical committee, in order to access the clinical history of the patient to make the present case report.

## 3 Clinical evaluation

A 1-year-old male patient, son of non-consanguineous parents and with no family history of skeletal dysplasias, who in the prenatal stage presented an anatomical size ultrasound at week 20 of gestation showing micromelia and pyelocalyceal dilation. At birth, the patient was found with body disproportion with shortening of the upper and lower limbs more proximal than distal, equinovarus foot, ligamentous laxity and dysmorphic facies, given by epicanthal folds, retrognathia, high palate, low-set lobed ears. Ophthalmological and auditory evaluation without alterations, at the cardiovascular level with transthoracic echocardiogram showing restrictive patent foramen ovale with aneurysmal septum primum to the right, normal LVEF systolic function 67 %. Long bones x-ray with preserved bone density, without traces of fractures or lytic or blastic lesions.

An X-ray of the upper limbs was performed, showing thickened and shortened long arm bones and forearms, without alteration of the hand bones. Ultrasound of the abdomen showed pyelocalyceal dilatation of the left kidney, bilateral hip ultrasound with grade II bilateral hip dysplasia, and X-ray of the lower limbs showed clubfoot, which was corrected with surgical management and a Dennis Brown splint.

## 4 Methods

To investigate the genetic causes of the clinical manifestations, we performed sequencing and deletion/duplication analysis on 358 candidate genes using genomic DNA obtained from both prenatal and postnatal samples, We used a protocol based on hybridization to enrich the genomic DNA obtained from submitted samples for target regions. We then used Illumina sequencing technology, which can identify individual bases as they are incorporated into DNA strands, to sequence the enriched DNA.^18^, ^19^

We sequenced all target regions at a depth of at least 50x, or performed additional analyses to ensure sufficient coverage. We aligned the resulting reads to the GRCh37 reference sequence and identified sequence changes in the context of a single clinically relevant transcript.

We focused our enrichment and analysis on the coding sequence of the indicated transcripts, as well as 10 bp of flanking intronic sequence (20 bp for BRCA1/2) and other specific disease-causing genomic regions identified at the time of assay design. We did not evaluate promoters, untranslated regions, or other non-coding regions, and for some genes, only specific target loci were analyzed.

We identified exonic deletions and duplications using an internal algorithm that compares the read depth of each target in the proband sequence to the mean read depth obtained from a set of clinical samples, in order to determine the number of copies in each target.

Markers on the X and Y chromosomes were analyzed for quality control purposes and to be able to detect deviations from the expected sex chromosome complement. All clinically significant observations were confirmed using additional testing methods, except for variants that were already confirmed in a first-degree relative or had been individually validated.

To analyzed the gen-gen interaction we used the GeneMANIA interface (https://genemania.org), in order to generated hypotheses about gene function, analyzed gene lists, and prioritized genes for functional assays, we obtained a network of genes associated with each other in the patient.^20^, ^21^

## 5 Result

Four pathogenic variants were obtained two heterozygous variants with pathogenic significance in *SLC26A*, one heterozygous pathogenic variant in *CEP120* and one heterozygous pathogenic variant in *CEP120*, Table 1.

**Table 1:** Genetic results.

***Gene***	**Variant**	**Zygosity**	**Classification**	**State**	**Associated**
*SLC26A2*	c.835C > T (p. Arg279Trp)	Heterozygous	Pathogenic	Affected	Spectrum of skeletal dysplasias with variable severity
*SLC26A2*	c.1343C > A (p. Ser448*)	Heterozygous	Pathogenic		
*CLCN7*	c.2049dup (p. Leu684Alafs*243)	Heterozygous	Pathogenic	Affected	Autosomal recessive osteopetrosis, autosomal dominant osteopetrosis and hypopigmentation, organomegaly, and delayed myelination and development.
*CEP120*	c.2164C > T (p. Arg722*)	Heterozygous	Pathogenic	Carrier	Joubert syndrome and short rib thoracic dysplasia

GeneMANIA interface led to obtain the network of genes associated with each other in the patient*,* Illustration. 1, in which 77.64 % physical interactions, 8.01 % co-expression, 5.37 % prediction, 3.63 % co-localization, 2.87 % genetic interactions, 1.88 % pathway of action and 0.60 % shared protein domains.

**Illustration. 1:**
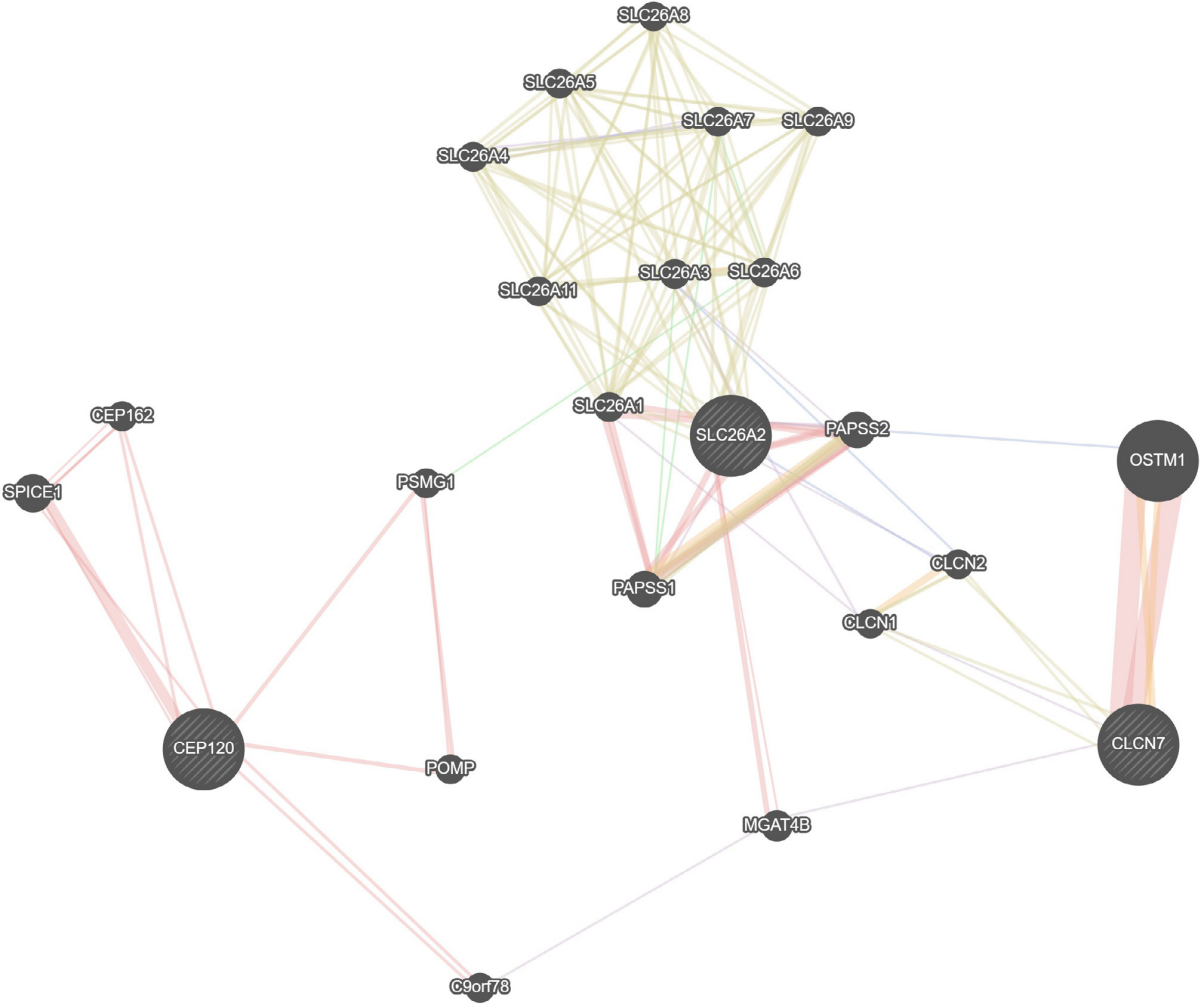
Gene expression networks associated with each other in the patient.

## 6 Discussion

SDs, also known as osteochondrodysplasias, are a group of heterogeneous disorders associated with bone and cartilage development; sometimes accompanied by pulmonary, genitourinary, visual, auditory, and neurodevelopmental manifestations. In 2019, the International Society for Skeletal Dysplasia published an updated version of the SD nosology, which includes 461 skeletal genetic disorders and identifies 437 different causative genes grouped into 42 categories based on their clinical, radiographic, and/or molecular characteristics. This comprehensive classification system highlights the challenges associated with diagnosing SDs due to their heterogeneous nature.^7^, ^9^, ^11^

SD are considered multiple congenital anomalies syndromes and have been linked to genes variants encoding components of RNA degradation, although we are currently unaware of a human disease caused by variants in a ribonuclease that specifically targets messenger RNA polyadenylation (poly(A)), which leads to the phenotype varying in severity. Based on an estimated incidence of 5 out of every 1000 pregnancies, it has been suggested that 75-–80 % of skeletal dysplasias detected prenatally are lethal.^8^, ^9^

A characteristic feature of the SD phenotype is body disproportion due to the shortening of the limbs, trunk, or both. If the limbs are short (short-limb dwarfism), linear growth deficiency may affect the proximal segment (humerus/femur), intermediate segment (radius and ulna/tibia and fibula), distal segment (hands/feet), or all the limb segments, the appropriate terms being, respectively, rhizomelic, mesomelic, acromelic shortening, or micromelia.^8^, ^9^

In recent decades, efforts have been made to elucidate the genetic pathophysiology of SD, which has allowed the emergence of new therapeutic approaches such as cell therapy, gene therapy, or drug therapy for various conditions. Several therapeutic strategies are currently being investigated in osteogenesis imperfecta. There are ongoing clinical trials based on pharmacological approaches, targeting signaling pathways in specific types of skeletal dysplasias, such as achondroplasia and Fibrodysplasia ossificans progressiva (FOP), or addressing endoplasmic reticulum stress in conditions such as Schmid-type metaphyseal dysplasia or pseudoachondroplasia. In addition, the treatment of hypophosphatasia, or Morquio A disease, illustrates the efficacy of enzyme drug replacement. These diseases require highly specialized and multidisciplinary approaches involving various specialists, such as geneticists, orthopedic surgeons, and endocrinologists. The emergence of treatments for skeletal dysplasia provides new insights into the prognosis of these severe conditions and may change prenatal advice for these diseases in the coming years.^11^

The patient described carried a compound heterozygous variant in the *SLC26A2* gene, also known as the “*solute carrier family 26 members 2*” gene, located in sub-band 2 of band 3 of the long arm of chromosome 5 (5q32). The *SLC26A2* gene encodes for a transmembrane glycoprotein that has several functions. It transports inorganic cations/anions and amino acids/oligopeptides across the cell membrane. It catalyzes the sulfonation of small molecules in the cytosol. It is involved in the activity of the secondary active sulfate transmembrane transporter and the transmembrane transporter of secondary active sulfate. It plays a role in the sulfation of proteoglycans in the extracellular matrix of the musculoskeletal system. Variations in the *SLC26A2* gene can affect the structure and organization of the extracellular matrix, leading to the development of the SD phenotype.^9^, ^22^, ^23^, ^24^, ^25^

The presented patient phenotype may clinically match with an intermediate form^26^ of diastrophic dysplasia (DTD; OMIM 222600) which previously described in association of compound heterozygous *SLC26A2* variants according to what has been previously described by Silveira et al (2023).^27^

The patient also had a pathogenic heterozygous variant in the *CLCN7* gene, which is also known as “*voltage-gated chloride channel 7*”. This gene is located in sub-band 3 of band 13 on the short arm of chromosome 16 (16p13.3) and encodes for the homonymous protein. The product of this gene belongs to the CLC chloride channel family of proteins. Chloride channels play an important role in the plasma membrane and intracellular organelles. Variations in the *CLCN7* gene are associated with different forms of osteopetrosis, including autosomal dominant osteopetrosis (ADO), autosomal recessive osteopetrosis (ARO), and the intermediate form. Defects in this gene are the cause of autosomal recessive osteopetrosis type 4 (OPTB4), also called infantile malignant osteopetrosis type 2, as well as the cause of autosomal dominant osteopetrosis type 2 (OPTA2), also known as autosomal dominant Albers-Disease, Schonberg disease, or autosomal dominant Marble disease. A variation in the *CLCN7* protein affects the function of osteoclasts, which are cells that break down bone tissue. This leads to impaired dissolution of the inorganic bone matrix and several clinical features associated with osteopetrosis.^28^, ^29^, ^30^, ^31^

The patient also carries a single heterozygous variant in the *CEP120* gene which is associated with recessive diseases and can confer carrier status for conditions known as ciliopathies. *CEP120* gene codes for the 120 kDa centrosomal protein, also known as coiled-coil 100 domain-containing proteins or gene that contains the coiled-coil 100 domain (CCDC100 gene), which is located in sub-band 2, thus band 23 of the long arm of chromosome 5 (5q23.2) and codes for the 120 kDa centrosomal protein, also known as coiled-coil 100 domain-containing proteins, which is required for centriole elongation from a procentriole which is essential for the formation of centrosomes, cilia, and flagella. Variations on the *CEP120* gene lead to Ciliopathies, which are a group of clinical disorders affecting the primary cilium, a 9 + 0 immobile monocilium, and may involve the mobile monocilium or the 9 + 2 motile cilia. These disorders include Joubert syndrome (JS), Jeune asphyxiant thoracic dystrophy (JATD), and short rib thoracic dysplasia.^32^, ^33^, ^34^, ^35^, ^36^, ^37^, ^38^, ^39^, ^40^

Even though kidney disease occurs in up to one third of patients with Joubert syndrome, most commonly in those with variations in *CEP120, CEP290*, *TMEM67*, and *AHI1*. Like other syndromic ciliopathies, the kidney disease can be very variable, going to mild to severe, but since the patient is a carrier, it is unlikely that these gene is the cause. Variations on *CLCN7* causes Hypopigmentation, organomegaly, and delayed myelination and development (OMIM: 618541) and autosomal dominant Osteopetrosis (OMIM: 166600) which causes organomegaly including enlargement of liver, kidney, and spleen and since the patient had pyelocalyceal dilation we could theorize that the kidney affection could be cause by the heterozygous pathogenic variant in *CLCN7*.^41^, ^42^, ^43^

Gen-gen interactions affects the cells by affecting the proteins. In normal gen-gen interactions proteins in the cell membrane interact with each other to affect the activity of transmembrane transporters, which are important groups of proteins that allow the transfer of a specific substance or a group of related substances, from one side of a membrane to the other. Cell membranes and organelle membranes are selectively permeable, meaning they only allow certain molecules to pass through, resulting in limited movement of molecules in and out of cells and organelles. Three main classes of transmembrane transporters have been described in the literature, all of which are integral transmembrane proteins and show a high degree of specificity for the transport of substances. The first class consists of pumps driven by ATP, also known as ATPases, which use the energy of ATP hydrolysis to move ions or small molecules through the membrane to resist gradients or chemical concentration potentials. Secondly, we have channel proteins that form a pore or channel in the membrane, allowing specific molecules or water to pass down their concentration or electrical potential gradients without requiring energy, which makes the process energetically favorable. The third class consists of transporters, which bind to specific molecules and move them across the membrane. This allows transporters to selectively transport specific molecules and maintain concentration gradients across the membrane.^44^, ^45^, ^46^ So when a gen variants interacts within each other it generates impaired transmembrane transportation.

GeneMANIA made it possible to show that genes typically do not act alone, but are part of a complex system called the genome, where they interact with other genes modifying the effects of a particular gene or genetic variant. These genetic interactions may be influenced by other genetic elements or external factors. In the present case, the interaction between these genes affects the activity of the inorganic anion exchanger, which normally allows the transfer of a solute or solutes from one side of a membrane to the other, depending on specific factors such as the type of reaction involved, as well as the activity of the transmembrane transporter of the sulfur molecular entity, which allows the transfer of a sulfur-containing molecule, such as sulfates or sulfites, from one side of a membrane to the other. The transmembrane dicarboxylic acid transporter moves dicarboxylic acids into, out of, or within cells or between cells, using a transporter or a pore. Transmembrane carboxylic acid transporter activity enables the transfer of organic acids containing carboxyl groups (COOH) or carboxylate anions (COO–) from one side of a membrane to the other. These compounds play a crucial role in skeletal health and their dysregulation has been linked to skeletal dysplasias and osteopetrosis. In these conditions, impaired carboxylic acid transport leads to disorganization of collagen fibers, early bone mineralization, and failure in the assembly of fibronectin from the extracellular matrix and also causing other organs affections, which leads to the patient intermediate and kidney phenotype.^21^, ^47^, ^48^, ^49^

## 7 Conclusions

Genetic interactions or epistasis play an important role in determining the risk, progression, and response to treatment of many diseases; therefore, identifying the interactions and how they affect the phenotypes of the different diseases becomes a challenge for current genomics because the variation of traits is determined not only by their genetic components, but also by how these genes interact with one another. Identifying these interactions within the genome can help us better understand the genetic architecture of complex traits, which in turn can lead to more personalized and precise treatments for many diseases. This is especially important because there is still much to learn about the heritability of many traits, and studying genetic interactions can help fill these gaps in our knowledge.

## Ethics approval and consent to participate

Not applicable to this case report.

## Consent for publication

Written and oral informed consent was obtained from patient and/or their legally authorized representative(LAR).

## Availability of data and materials

All data generated or analysed during this study are included in this published article.

## Funding

No funding was received for the redaction of the case report.

## Authors' contributions

Each author contributed to the redaction, proofreading, and correction of the manuscript. NYM contributed to the research, writing, and proofreading, while LJNM contributed in corrected and adding relevant medical changes to the case. All authors read and approved the final manuscript. All authors participated in the acquisition, analysis, and interpretation of the data. Each author has agreed both to be personally accountable for their contributions and to ensure that questions related to the accuracy or integrity of any part of the work, (even ones in which the author was not personally involved), are appropriately investigated, resolved, and the resolution documented in the literature. All authors read and approved the final manuscript.

## Credit authorship contribution statement

**Nathalie Yepes Madrid:** Investigation, Writing – original draft, Writing – review & editing.

## Declaration of competing interest

The authors declare that they have no known competing financial interests or personal relationships that could have appeared to influence the work reported in this paper.
